# Quantitative Proteomic Analysis Reveals Key Proteins Involved in Testicular Development of Yaks

**DOI:** 10.3390/ijms25158433

**Published:** 2024-08-02

**Authors:** Yongfu La, Xiaoming Ma, Pengjia Bao, Min Chu, Ping Yan, Xian Guo, Chunnian Liang

**Affiliations:** 1 Key Laboratory of Animal Genetics and Breeding on Tibetan Plateau, Ministry of Agriculture and Rural Affairs, Lanzhou 730050, China; layongfu@yeah.net (Y.L.); 82101171175@caas.cn (X.M.); baopengjia@caas.cn (P.B.); chumin@caas.cn (M.C.); pingyanlz@163.com (P.Y.); 2 Key Laboratory of Yak Breeding Engineering, Lanzhou Institute of Husbandry and Pharmaceutical Sciences, Chinese Academy of Agricultural Sciences, Lanzhou 730050, China

**Keywords:** yak, testis, reproduction

## Abstract

Male reproductive health is largely determined already in the early development of the testis. Although much work has been carried out to study the mechanisms of testicular development and spermatogenesis, there was previously no information on the differences in the protein composition of yak testicles during early development. In this study, the protein profiles in the testicles of 6- (M6), 18- (M18), and 30-month-old (M30) yaks were comparatively analyzed using TMT proteomics. A total of 5521 proteins were identified, with 13, 1295, and 1397 differentially expressed proteins (DEPs) in 30- vs. 18-, 18- vs. 6-, and 30- vs. 6-month-old testes, respectively. Gene Ontology (GO) annotation and Kyoto Encyclopedia of Genes and Genomes (KEGG) enrichment analysis showed that DEPs were mainly involved in signaling pathways related to testicular development and spermatogenesis, including the MAPK, PI3K–Akt, Wnt, mTOR, TGF-β, and AMPK signaling pathways. Furthermore, we also identified eight potential proteins (TEX101, PDCL2, SYCP2, SYCP3, COL1A1, COL1A2, ADAM10, and ATF1) that may be related to the testicular development and spermatogenesis of yaks. This study may provide new insights into the molecular mechanisms of the testicular development and spermatogenesis of yaks.

## 1. Introduction

Yak is an important domesticated species on the Qinghai–Tibet Plateau, with good adaptability to the alpine climates [1]. More than 16 million yaks provide a large amount of daily necessities, including meat, milk, fuel, and hides for nomadic herders living in high-altitude areas [2,3,4]. In a breeder herd, one male yak is responsible for fertilizing dozens of female yaks. Therefore, the fertility of the male yak is one of the first limiting factors to achieving the highest reproduction possible. The testicle is the most important reproductive organ in males; it can secrete sex hormones and produce sperm. Testicular development includes the normal development of spermatogenic cells, Sertoli cells, and Leydig cells. Research shows that early testicular development determines the reproductive ability of male animals for a lifetime [5,6,7]. Therefore, using omics techniques to explore the molecular mechanisms of testicular development in yaks can effectively improve the reproductive performance of male yaks.

Gene and mRNA levels do not respond well to physiological changes due to pre- and post-transcriptional regulation [8]. Proteomics technology is a powerful tool for analyzing complete protein composition through post-transcriptional analysis [9]. Tandem mass tag (TMT) technology is a high-throughput screening method for quantitative proteins, widely used for measuring changes in protein expression levels under different physiological conditions [10,11]. To date, proteomics technology has been successfully used in testicular development research [12,13,14]. However, no reports had previously been found on the changes in protein levels in the testicular tissues of yaks at different developmental stages.

In this study, we used proteomic techniques to analyze the changes in protein expression levels of yak testicular tissue at different developmental stages. Our research aims to identify the differences in the teste’s proteomes of yaks at different developmental stages and reveal key proteins that may play an important role in improving male yak reproductive performance.

## 2. Results

### 2.1. Testicular Development and Histology

**Figure 1:**
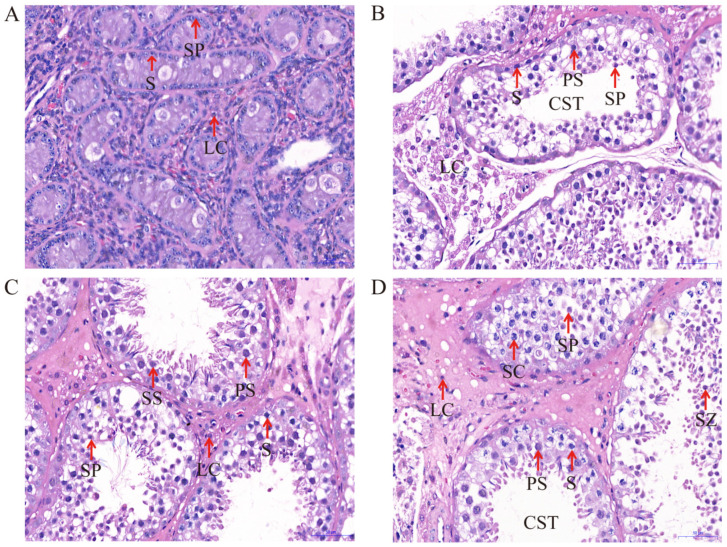
H&E staining of yak testicular tissues at different developmental stages (400×): (**A**) 6 months of Ashidan yak; (**B**) 18 months of Ashidan yak; (**C**) 30 months of Ashidan yak; (**D**) 72 months of Ashidan yak; Seminiferous tubule (CST); Sertoli cells (SC); Leydig cells (LC); Spermatogonia (S); Primary spermatocytes (PS); Secondary spermatocytes (SS); Spermatids (SP); Spermatozoon (SZ).

The histological findings at different developmental stages of yak testes obtained through H&E staining are shown in Figure 1. The results show that capillaries, myoid cells, Leydig cells, Sertoli cells, spermatogonia, round spermatids, and primary spermatocytes are present in all groups (6, 18, 30, and 72 months), while the tubular compartment constitutes the majority of the testicular parenchyma. We found that the cross-sectional area, volume density, and epithelial thickness of both seminiferous epithelium and seminiferous tubules gradually increase with the age of animals. As shown in Table 1, compared with 6 and 18 months, the short and long diameters of the seminiferous tubules in the testis increased significantly at 30 and 72 months (*p* < 0.05). This difference was not significant between 6 and 18 months, nor between 30 and 72 months. The short and long diameters of the spermatogonium, spermatocyte, and Leydig cells in the different groups were not significant (*p* > 0.05). In addition, compared with the 18-month-old group, the short and long diameters of the Sertoli cells were significantly larger (*p* < 0.05) in the testes of other groups.

### 2.2. Comparison of Protein Profiles

**Figure 2:**
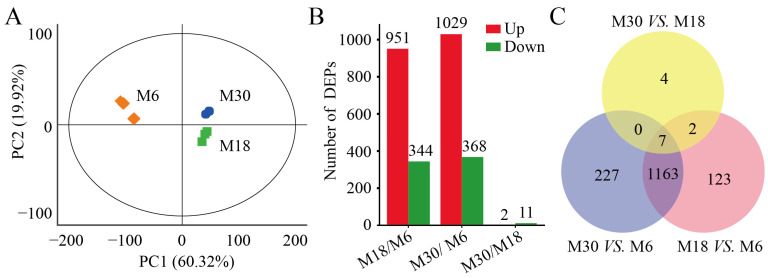
Comparison of protein profiles in the testes of yaks: (**A**) principal component analysis (PCA) of the proteome in the testes of the three groups of yaks; (**B**) distribution of differentially expressed proteins (DEPs) in the testes of the three groups of yaks; and (**C**) Venn diagram of DEPs in pairwise comparisons of the three types of yak testes.

In this study, a total of 6131 proteins were identified from 6-, 18-, and 30-month-old yak testes, and 5521 proteins were quantifiable proteins. The principal component analysis (PCA) was used to compare the proteomes of yak testicular tissue at different developmental stages, and the results show differences between the M6, M18, and M30 testicular proteomes (Figure 2A).The proteins of the three yak testes were separated in PCA, with principal component 1 (PC1) and principal component 2 (PC2) values of 60.32% and 19.92%, respectively. The distribution of PC1 dimensions shows that M6 testis proteins were significantly separated from M18 and M30 testes proteins, which indicates that the physiological activities of yak testis may be different before and after sexual maturity.

Differential expression analysis of quantifiable proteins was performed using fold changes ≥ 1.5 and *p* < 0.05 between any two groups, and a total of 1526 DEPs were obtained. Consistent with the results of the PCA, the highest number of DEPs, 1397 were found between M30 and M6, followed by 1295 found between M18 and M6, and the lowest number, 13 found between M30 and M18 (Figure 2B). In detail, 1029 were upregulated and 368 were downregulated between M30 and M6 (Table S1); 951 were upregulated and 344 were downregulated between M18 and M6 (Table S2); 2 were upregulated and 11 were downregulated between M30 and M18 (Table S3). Most of these DEPs were revised upward. Only seven DEPs overlapped in the two-by-two comparison between the three periods of yak testicular tissue proteomes (Figure 2C). These seven DEPs, which were coexpressed in the testes of yaks at three development stages, may be the key proteins for yak testicular development and spermatogenesis.

### 2.3. GO Analysis of DEPs

**Figure 3:**
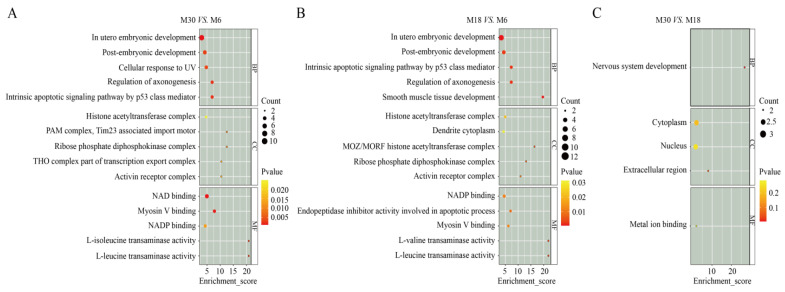
Gene ontology enrichment analysis of differentially expressed proteins: (**A**) GO enrichment analysis of differentially expressed proteins between M30 and M6. (**B**) GO enrichment analysis of differentially expressed proteins between M18 and M6. (**C**) GO enrichment analysis of differentially expressed proteins between M30 and M18.

We explored the physiological functions of the proteins involved in the testicular tissue of yaks at 6, 18, and 30 months old through GO annotation and classification. There were 1397, 1295, and 13 DEPs annotated in the M30 vs. M6, M18 vs. M6, and M30 vs. M18 groups, respectively. As shown in Figure 3, through protein functional analysis, the DEPs were enriched in three categories of GO terms, including biological process, cellular component, and molecular function. Between M30 and M6, the top three GO terms that were significantly enriched in biological processes were the cellular response to menadione, the branched-chain amino acid biosynthetic process, and the in utero embryonic development (Figure 3A and Table S4). Between M18 and M6, the top three GO terms that were significantly enriched in biological processes were the in utero embryonic development, the smooth muscle tissue development, and the positive regulation of blood vessel endothelial cell proliferation involved in sprouting angiogenesis (Figure 3B and Table S5). Between M30 and M18, the top three GO terms that were significantly enriched in biological processes were the regulation of synaptic vesicle recycling, the L-threonine catabolic process to glycine, and the AMP biosynthetic process (Figure 3C and Table S6).

### 2.4. KEGG Analysis of DEPs

The KEGG pathway annotation of DEPs in M30 vs. M6 and M18 vs. M6 shows that they were involved in 297 and 284 signaling pathways, respectively, including 16 signaling pathways related to male reproductive function (Table 2). However, the KEGG pathway annotation of DEPs in M30 vs. M18 revealed that they only involved four signaling pathways, including the Glycine, serine and threonine metabolism; the Pentose phosphate pathway; the Purine metabolism; and natural killer cell-mediated cytotoxicity (Tables S7–S9).

### 2.5. Validation of TMT Data for Selected Proteins by PRM

In this study, we selected some proteins related to male reproductive health for PRM analysis to verify the results of TMT. The PRM analysis showed that the protein expression trend detected by PRM was basically consistent with the TMT results (Table 3). However, the differences between the actual values may be caused by different detection methods. Therefore, our PRM analysis indicates that TMT data have high credibility and could be used for further analysis.

## 3. Discussion

In male mammals, proteins in the testes play a crucial role in male hormone synthesis and spermatogenesis [15]. However, during the early development process of male animals, changes in several factors can affect the efficiency of gonadal function, thereby determining the reproductive performance of male animals [16]. Therefore, studying the histological characteristics and proteomic changes in the testes during the early development of yaks is crucial for elucidating the regulatory mechanisms of efficient reproductive performance in yaks. In recent years, several studies have compared the proteomic and histological characteristics of the testes in pigs, sheep, cattle, and mice at different developmental stages [17,18,19]. In this study, the proteomic and histological characteristic changes during the development of yak testes were studied. The histological analysis showed that the cross-sectional area of the seminiferous tubules increased during the development of yak testes, indicating the expansion of the seminiferous tubules and various testicular cells (Leydig, germ, and Sertoli cells) during testicular development [20,21,22]. Then, the comparative TMT proteomics method was used to detect the proteome of three different developmental stages of yak testes. In total, 1397, 1295, and 13 differentially expressed proteins were identified in M30 vs. M6, M18 vs. M6, and M30 vs. M18, respectively, in which 7 were common proteins differentially expressed at the 6-, 18-, and 30-month stages. Next, we analyzed the functions of these differentially expressed proteins, elucidated the expression patterns of proteins in yak testes at different developmental stages, and explored proteins related to regulating yak testicular development and spermatogenesis.

In this study, there are several proteins worth mentioning that are upregulated in the M30 and M18 groups and participate in male reproduction. TEX101 is a glycoprotein molecule specifically expressed during germ cell meiosis and in testicular sperm [23]. TEX101 plays a molecular chaperone role during fertilization and is crucial in the production of functionally intact sperm [24]. Knocking out TEX101 leads to infertility in male mice [25]. PDCL2 is a germline-specific gene belonging to the phoducin family and is crucial for sperm acrosome development and male reproductive performance [26,27]. A functional deficiency of the PDCL2 gene causes abnormal acrosome biogenesis during spermiogenesis and the loss of sperm motility, leading to infertility [28]. SYCP2 and SYCP3 are the main components of the lateral element, which forms a synaptonemal complex (SC) with protein aggregates composed of a central region composed of intermediate central elements with a width of 20–40 nm [29]. A previous study showed that SYCP2, as an important transcription regulatory factor, can interact with lncRNA to regulate spermatogenesis [30]. SYCP3 is specifically expressed in human testicular germ cells, and a lack of SYCP3 expression may have a negative impact on spermatogenesis and male fertility [31]. In this study, the expression of TEX101, PDCL2, SYCP2, and SYCP3 was significantly higher in M18 and M30 yak testes than in M6 yak testes, indicating that these genes may have important regulatory effects on spermatogenesis in yak testes.

We also found that some downregulated proteins were related to male reproduction. The COL1A1 and COL1A2 genes encode procollagen I, which is composed of α2 and two α1 chains; it plays important regulatory roles in cell proliferation and differentiation. Studies have shown that COL1A1 and COL1A2 mediate the separation and migration of germ cells during spermatogenesis [32]. ADAM10 is a metalloproteinase expressed in male germ and Sertoli cells, playing an important role during the apoptosis process of germ and Sertoli cells [33]. ATF1 belongs to the CREB/ATF transcription factor family and plays an important regulatory role in the production of sperm and male germ cell proliferation [34]. In this study, the expression of COL1A1, COL1A2, ADAM10, and ATF1 proteins was downregulated in M18 and M30, indicating that these genes may be involved in regulating yak testicular development and spermatogenesis. At the same time, histological sections of yak testicles showed an increase in cell types and numbers in M18 and M30, which may be the cause of these proteins’ regulation of testicular cell proliferation and differentiation.

Pathway enrichment analysis in KEGG revealed that some of the identified DEPs were involved in the MAPK, PI3K-Akt, Wnt, mTOR, TGF-β, AMPK, ribosome, insulin resistance, and insulin signaling pathways that are related to spermatogenesis and testicular development. The MAPK, AMPK, and TGF-β signaling pathways are involved in the spermatogenesis, testicular cell proliferation, and functional regulation of mature Sertoli and Leydig cells [35,36]. Compared with 6-month-old yaks, some proteins from the MAPK, AMPK, and TGF-β pathways are upregulated in the testicles of 18- and 30-month-old yaks, indicating that these proteins may play an important role in promoting the testicular development and spermatogenesis of yaks. In the testes, KIF17 mediates the transmission of ACT from the nucleus to the cytoplasm and the transport of Spatial-ε, thereby regulating spermatogenesis [37]. INSL6 is an insulin/relaxin family peptide hormone primarily expressed in male germ cells [38]. A gene knockout experiment demonstrated that INSL6 is necessary for the process of spermatogenesis and that INSL6 knockout results in spermatogenic failure [39]. Transcription elongation factor A2 (TCEA2), also known as TFIIS, is a stimulating protein of RNA polymerase II specifically expressed in testicular germ cells [40]. A recent study showed that TCEA2 is only expressed in spermatocytes and not significantly expressed in spermatids, spermatogonia, nor Leydig cells [41]. It was found in mice that TCEA2 is a specific transcriptional elongation factor necessary for spermatogenesis [42]. In this study, the number of spermatogonia in the testes of M18 and M30 yaks increased, and the presence of sperm cells was found, indicating that the spermatogenesis process of yaks may be regulated by KIF17, INSL6, and TCEA2.

Additionally, some studies have confirmed the activity of PI3K-Akt, Wnt, and mTOR signaling in mice, cattle, and sheep testes [43,44]. Among them, the PI3K-AKT signaling pathway is one of the most important regulatory mechanisms in animal cells, mainly regulating cell line anti-apoptosis, survival, and proliferation. The Wnt signaling pathway plays an important role in the differentiation of Sertoli cells, and the disruption of Wnt signaling leads to apoptosis and the rapid loss of germ cells [45]. It has been reported that the mTOR signaling pathway plays a role in the proliferation and differentiation of testicular cells and spermatogenesis [46]. These studies indicate that PI3K-Akt, Wnt, and mTOR signaling are crucial for testicular development and spermatogenesis. However, their regulatory mechanisms for testicular development and spermatogenesis in yaks require further research. We found that the regulatory mechanism of male yak reproduction is related to the regulation of the PI3K-Akt, Wnt, and mTOR signaling pathways. These results provide a reference for research on the regulatory mechanisms of testicular development and spermatogenesis in yaks.

## 4. Materials and Methods

### 4.1. Ethics Statement

The care of the animals was in accordance with the National Institutes of Health guidelines, and all procedures were approved by the Animal Administration and Ethics Committee of the Lanzhou Institute of Husbandry and Pharmaceutical Sciences of the Chinese Academy of Agricultural Sciences (Ethics Approval Code: 2019–002).

### 4.2. Animals and Sample Preparation

All animals used in this study were from nucleus herds of Ashidan yaks in the Da-tong Breeding Farm of Qinghai province. The nine selected Ashidan yaks from different families were healthy and fed in an outdoor setting under similar conditions of temperature, illumination, and nutrition level. Furthermore, the animals were divided into three groups, namely 6- (M6), 18- (M18), and 30-month-old (M30) male yaks. Each group contained three male yaks. The nine male yaks were slaughtered, and tissues from their left testes were collected. Each testis was separated into two parts from the center: one part of the sample was immediately frozen in liquid nitrogen and stored at −80 °C until further analysis; from the other part, a tissue sample was collected with a volume of 1 cm^3^ (10 mm × 10 mm × 10 mm) from the center and fixed in 4% formalin buffer for 48 h prior to paraffin embedding and stored for histological examination.

### 4.3. Testicular Histomorphology

Fixed yak testicle samples were cut into sections of 5 μm thickness, mounted on glass slides, and stained with hematoxylin and eosin for light microscopy. A BA200Digital digital microscope (Motic China Group Ltd., Xiamen, China) was used to capture the photomicrographs. Take 20 cross-sectional images (40× and 400× objective lenses) of the roundest seminiferous tubes for each sample, and measure the diameter and radius of the seminiferous tubes simultaneously. The mean value was obtained by measuring the orthogonal positions of two seminiferous epithelium heights. For Leydig cells, 10 sections were detected in each sample and analyzed using 400× objective lens and image analysis software (Sunny, Ningbo, China).

### 4.4. TMT Proteomic Analysis

The total protein content was extracted from 9 yak testes of the M6, M18, and M30 groups using the SDT (4% [*w*/*v*] SDS, 100 mM Tris/HCl pH 7.6, 0.1 M DTT) lysis method, and was quantified by the BCA method. As described by Huang et al. [47], protein samples were labeled with TMT and the labeled samples were subjected to reverse-phase nanoflow liquid chromatography tandem mass spectrometry as previously described [48]. In the Bos_grunniens.LU_Bosgru_v3.0.pep.all. fasta database, peptide identification and quantification was conducted using Mascot 2.2 and Proteome Discover 2.4 search results data (Thermo Fisher Scientific, Carlsbad, CA, USA). Next, perform quantitative data analysis on proteins with Score Sequest HT > 0 and unique peptide ≥ 1. A *t*-test was used to compare the differences in protein expression between each two comparison groups and calculate the *p*-value. Proteins with expression fold changes ≥ 1.5 and *p*-values < 0.05 were considered to be significantly differentially expressed.

### 4.5. Protein Functional Annotation and Enrichment Analysis

In the process of studying the functions of the differentially expressed proteins obtained, we used GO annotation software to annotate the functions of differentially expressed proteins obtained from all comparison groups [49]. Secondly, we conducted KEGG annotation analysis on differentially expressed proteins using the KEGG Automatic Annotation Server (KAAS) and the Kyoto Encyclopedia of Genes and Genomes (KEGG) database (http://www.genome.jp, accessed on 21 October 2022) [50]. Fisher’s exact test was used to test the differentially expressed proteins. The calculated *p*-value was corrected by Benjamin and Hochberg, with a corrected *p*-value ≤ 0.05 as a threshold.

### 4.6. Protein Validation by 4D-PRM

As a technology, 4D-PRM is used for verifying the quantified proteins through unique peptides. In this study, we selected 10 highly abundant differentially expressed proteins from the TMT-MS analysis for validation by 4D-PRM (Oebiotech Co., Ltd., Shanghai, China). The PRM analysis used samples from proteomics analysis and defined characteristic peptides of target proteins based on TMT data. Perform protein extraction and trypsin digestion as described above. Dissolve the peptides in mobile phase A (99.9% H_2_O, 0.1% FA) and separate using the NanoElute liquid-phase system (Bruker Co., Ltd., Billerica, MA, USA, GER). After UPLC separation, the peptides were injected into a capillary ion source for ionization and then analyzed using a Tims-TOF Pro2 mass spectrometer.

### 4.7. Statistical Analysis

The data were analyzed using one-way ANOVA with SPSS 21.0 software (SPSS, Chicago, IL, USA). The data are represented as “means ± SD”, and *p* < 0.05 is considered statistically significant for the difference.

## 5. Conclusions

This study represents the initial comprehensive examination of the proteomic alterations in the testicles of yaks at different developmental stages, revealing that testicular development and spermatogenesis in yaks are essentially multifactorial. Furthermore, through proteomic analysis, we identified some key proteins related to the testicular development and spermatogenesis of yaks. These proteins are mainly related to the MAPK, PI3K-Akt, Wnt, mTOR, TGF-β, and AMPK signaling pathways. The results of this study contribute to elucidating the regulatory mechanisms of testicular development and spermatogenesis and provide valuable biomarkers for improving the reproductive performance of male yaks.

## Figures and Tables

**Table 1:** Different month-old yak testicular seminiferous tubule, spermatogonium, spermatocyte, Sertoli cell, and Leydig cell diameters (Unit: μm).

Types	Months	Rete Tubules	Spermatogonia	Spermatocytes	Sertoli Cells	Leydig Cells
Short diameter	6	43.75 ± 2.343 ^a^	4.02 ± 0.522	6.31 ± 0.891	3.17 ± 0.399 ^a^	4.37 ± 0.578
	18	56.30 ± 6.151 ^b^	3.86 ± 0.300	5.40 ± 0.415	2.97 ± 0.562 ^b^	4.47 ± 1.148
	30	140.16 ± 11.678 ^a^	4.08 ± 0.548	5.63 ± 0.715	5.01 ± 0.449 ^c^	4.14 ± 0.791
	72	167.564 ± 8.980 ^a^	4.57 ± 0.839	5.58 ± 1.126	4.67 ± 0.729 ^d^	8.47 ± 0.942
Long diameter	6	57.76 ± 8.299 ^a^	4.96 ± 0.405	6.46 ± 0.686	7.33 ± 0.833 ^a^	5.40 ± 1.235
	18	81.63 ± 6.285 ^b^	4.78 ± 0.911	6.85 ± 0.639	5.57 ± 0.562 ^b^	6.04 ± 1.537
	30	226.62 ± 47.394 ^a^	5.57 ± 0.716	6.59 ± 0.449	7.97 ± 0.341 ^c^	7.31 ± 2.459
	72	209.84 ± 26.630 ^a^	5.59 ± 0.321	7.02 ± 1.357	7.07 ± 0.939 ^c^	6.23 ± 0.754

Note: Different letters for the groups indicate differences (*p* < 0.05).

**Table 2:** DEPs involved in male reproduction-related signaling pathways.

Map_ID	Map_Name	Test_Seq
ko04010	MAPK signaling pathway	KIF17, INSL6, FAM136A, ERK2, JNK3, SHCBP1L, REEP6, ZNF474, MCM5, LACTB2, TCEA2, YWHAQ, PRAK
ko04151	PI3K-Akt signaling pathway	ACRBP, SEPTIN2, PRO768, GPR161, BAG6, FAM71B, ERK2, TMEM5
ko04910	Insulin signaling pathway	KIF17, RALGPS2, CCT2, GPR161, CCDC167, JNK3, ERK2, IRF2BPL
ko04310	Wnt signaling pathway	GAR1, CFL2, JNK3, LTF, GSTO1, R3HCC1, PEDF, NHLRC3
ko04931	Insulin resistance	SEPTIN2, CCDC167, TUBG2, JNK3, IRF2BPL, RAB3D
ko03010	Ribosome	MACROH2A1, FAM175B, PDIA5, SNU13, TUSC3, ACE, KIF5A, CCNL2
ko04150	mTOR signaling pathway	KIF17, GAR1, GPR161, R3HCC1, FEM1A, ERK2
ko04350	TGF-beta signaling pathway	UBAP2, TBC1D23, GFPT2, ERK2, YWHAQ
ko04140	Autophagy-animal	GPR161, JNK3, TRIM36, RNF146, ERK2
ko03008	Ribosome biogenesis in eukaryotes	CALD1, STK33, FAM32A, DAZAP1
ko01522	Endocrine resistance	KIF17, LIN7C, JNK3, ERK2
ko04152	AMPK signaling pathway	SEPTIN2, IRF2BPL, GPR161, CCDC167
ko00520	Amino sugar and nucleotide sugar metabolism	DNAJB1, RALGPS2, ESX1
ko04662	B cell receptor signaling pathway	ERK2, GTSF1, FAM136A
ko04371	Apelin signaling pathway	ERK2, YWHAQ
ko00100	Steroid biosynthesis	FBXW9

**Table 3:** Confirmation of DEPs detected in TMT analysis by 4D-PRM analysis.

Description (NCBI)	Gene Name	Fold Change (TMT)	Fold Change (4D-PRM)
Acrosin binding protein	ACRBP	4.36	1.89
Retinoic acid receptor responder 2	RARRES2	4.54	2.04
Synaptotagmin like 2	SYTL2	5.03	2.13
LYR motif containing 1	LYRM1	5.26	2.07
Protein disulfide isomerase like, testis expressed	PDILT	3.89	1.68
Phosducin like 2	PDCL2	3.79	1.37
Tyrosylprotein sulfotransferase 2	TPST2	0.32	0.62
ADAM metallopeptidase domain 10	ADAM10	0.50	0.44
STAG3 cohesin complex component	STAG3	0.45	0.29
Storkhead box 1	STOX1	0.24	0.27
Fascin actin-bundling protein 1	FSCN1	0.39	0.08
Mitogen-activated protein kinase kinase 1	MAP2K1	0.51	0.38

## Data Availability

Data is contained within the article and Supplementary Materials.

## Supplementary Materials

The following supporting information can be downloaded at: https://www.mdpi.com/article/10.3390/ijms25158433/s1.

## Abbreviations

DEPs: differentially expressed proteins; GO: Gene Ontology; KEGG: Kyoto Encyclopedia of Genes and Genomes; M6: 6-month-old; M18: 18-month-old; M30: 30-month-old; M72: 72-month-old.
